# A global map of terrestrial habitat types

**DOI:** 10.1038/s41597-020-00599-8

**Published:** 2020-08-05

**Authors:** Martin Jung, Prabhat Raj Dahal, Stuart H. M. Butchart, Paul F. Donald, Xavier De Lamo, Myroslava Lesiv, Valerie Kapos, Carlo Rondinini, Piero Visconti

**Affiliations:** 1 Ecosystems Services and Management Program (ESM), International Institute for Applied Systems Analysis (IIASA), Schlossplatz 1, A-2361 Laxenburg, Austria; 2 Global Mammal Assessment Program, Department of Biology and Biotechnologies, Sapienza University of Rome, Viale dell’Università 32, 00185 Rome, Italy; 3 BirdLife International, David Attenborough Building, Pembroke Street, Cambridge, CB2 3QZ UK; 4 Department of Zoology, University of Cambridge, Downing Street, Cambridge, CB2 3EJ UK; 5 UN Environment World Conservation Monitoring Centre (UNEP-WCMC), 219 Huntingdon Road, Cambridge, CB3 0DL United Kingdom

**Keywords:** Biogeography, Macroecology, Environmental sciences, Biodiversity

## Abstract

We provide a global, spatially explicit characterization of 47 terrestrial habitat types, as defined in the International Union for Conservation of Nature (IUCN) habitat classification scheme, which is widely used in ecological analyses, including for quantifying species’ Area of Habitat. We produced this novel habitat map for the year 2015 by creating a global decision tree that intersects the best currently available global data on land cover, climate and land use. We independently validated the map using occurrence data for 828 species of vertebrates (35152 point plus 8181 polygonal occurrences) and 6026 sampling sites. Across datasets and mapped classes we found on average a balanced accuracy of 0.77 ($\bar{+}$0.14 SD) at Level 1 and 0.71 ($\bar{+}$0.15 SD) at Level 2, while noting potential issues of using occurrence records for validation. The maps broaden our understanding of habitats globally, assist in constructing area of habitat refinements and are relevant for broad-scale ecological studies and future IUCN Red List assessments. Periodic updates are planned as better or more recent data becomes available.

## Abstract (summary)

**Table:** 

Measurement(s)	IUCN habitat/species management area
Technology Type(s)	Geographic Information System • digital curation • computational modeling technique
Sample Characteristic - Environment	terrestrial natural environment • anthropogenic terrestrial biome
Sample Characteristic - Location	Earth (planet)

Machine-accessible metadata file describing the reported data: 10.6084/m9.figshare.12619283

## Background & Summary

Habitat loss is one of the primary causes of biodiversity decline^1–4^. There are many definitions of ‘habitat’, but they can broadly be described as the entirety of the physical conditions - including land cover and climate - that enable a species’ population to persist in space and time^5^. There is a strong positive relationship between the extent and intactness of a species’ habitat and its population persistence^6–8^, which may help species extinction risk assessments when information about other symptoms of risk is limited. Knowledge about species’ habitats is critical to design landscape management plans^9^, conservation planning^10,11^ and analysis of past trends and future scenarios of species’ extinction risk^12–14^.

There are many ways to delimit species’ habitats types^15–17^, which can be represented as either continuous variables^17,18^ or discrete classes^19^. The International Union for Conservation of Nature (IUCN) Red List of Threatened Species uses a global standard typology (https://www.iucnredlist.org/resources/habitat-classification-scheme) that aims to categorize all species-relevant habitats into a system of pre-defined habitat classes^16^. In this scheme 16 different broad habitat classes are listed at level 1 (e.g. forest, wetlands), with 119 more specific classes listed at level 2 (e.g. Forest – Subtropical/tropical moist lowland). Although detailed descriptions of the habitat classes in this classification scheme are unfinished - with the latest available documentation draft dating to December 2012 - it is used by IUCN Red List assessors to describe species’ habitats preferences^20^.

IUCN Red List assessments also involve compiling distribution maps showing the range boundaries for each species, typically based on point locality data, presence/absence data from atlases, published maps in field guides and monographs, remote sensing data on habitat extent, and expert inference (e.g.^20–22^). Such maps are typically used to estimate Extent of Occurrence (the area of a minimum convex polygon that contains all occurrence records) in Red List assessments, and are also used in aggregate to quantify spatial biodiversity patterns at regional and global scales^23^. However, maps showing distributional boundaries often considerably overestimate the occurrence of a species at finer scales^11,24^, a type of error commonly known as commission error. To obviate these types of errors, one approach is to use the habitat preferences and elevational range documented in IUCN Red List assessments to exclude all land-cover classes and altitudes that are not considered suitable for a species in order to map its ‘Area of Habitat’ (AOH,^20^). This requires a ‘crosswalk’ that establishes the relationships between each habitat and land-cover class in a particular land-cover product^13,25,26^. However establishing such relationships between different thematic legends can be problematic.

Differences in thematic resolution and definitions can lead to large variations in area-based land-cover estimates^27^, and errors have been shown to increase uncertainty and decrease accuracy of any subsequent analysis^28^. These problems are likely to affect AOH estimates as described above, for instance by treating climatically distinct habitats - such as savannah-dominated and subtropical-moist shrub-covered land - as equivalent. Although the potential distribution of a species can be estimated statistically^29,30^, it is challenging to do so in a robust, consistent and reproducible manner^31,32^ and in most cases the primary biodiversity data necessary to do so are not available^33^. There is therefore a need to explore alternative approaches to mapping AOH.

Here we describe a method to map the IUCN habitats classification scheme directly for most terrestrial and inland water habitats. We do so by overlaying the best available data on land cover, climate and other ancillary data sources using simple map algebra. The derived map describes the global distribution of habitats at levels 1 and 2 as outlined by the IUCN classification scheme in the year 2015^16^. We validated the classes from this global map using independent spatially-explicit estimates. To our knowledge this is the first attempt to map IUCN habitat classes at a global scale.

## Methods

**Fig. 1:**
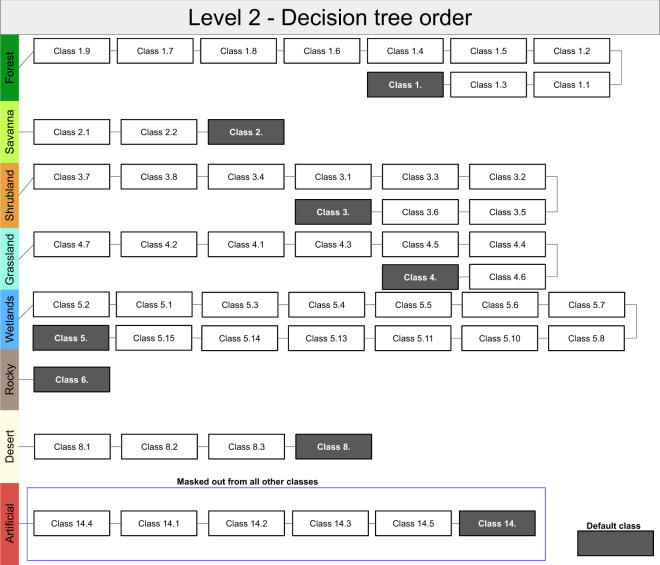
Sequential order in which habitat classes were identified using our decision tree approach. For instance, if the conditions do not match for IUCN habitat class 1.9, then the conditions for class 1.7 are tested afterwards. Black boxes indicate default classes (Level 1 code) in case no conditions could be met at level 2. Artificial habitat classes (blue border) are masked out from all other habitat classes. Codes and rulesets for each habitat class are further explained in Supplementary Table 1.

We delineated terrestrial habitat classes following the IUCN classification scheme by intersecting data on land cover, climate and land use. This intersection was done following a decision tree approach (Fig. 1), i.e. if the conditions for class 1.9 (Forest – Subtropical/tropical moist montane) were not true for a grid cell then class 1.7 (Forest – Subtropical/tropical mangrove vegetation) was tested. Thus each grid cell of the habitat map is allocated to a single IUCN habitat class. For global land cover, we used the Copernicus land-cover product^34^, which has 23 thematic classes at a ~100 m resolution and an overall average accuracy of ~80%. We used the discrete land cover classification as well as the Copernicus fractional forest cover estimates available for the year 2015. For climate, we used data on the world’s climatic zones based on the global Köppen-Geiger climate classification system^35^ at approximately 1 km resolution for the present climate (climatology 1980–2016). We also used the distribution of some terrestrial ‘biomes’^36,37^ for additional fine adjustment of climatic zones and to create a global mask of the subtropics & tropics.

In addition, we also considered a number of ancillary data layers for predominantly natural and anthropogenically defined habitat classes (Supplementary Table 1). To separate lowland and mountainous habitat classes, we used the ‘K1’ global mountain mask^38^, as well as elativation data from the Shuttle Radar Topography Mission (SRTM) mission at ~90 m resolution^39^. For IUCN wetland habitat classes - which follow the Ramsar Wetland type classification system^16^ - we used the Global Lakes and Wetlands Database (GLWD) at ~1 km resolution^40^, which we expanded with a ~5 km modal filter to account for small-scale differences in water cover (compared to Copernicus). For seasonal and intertidal wetlands and lakes we also considered information from HydroLAKES and other remotely-sensed water surface data^41–43^.To represent tropical and subtropical swamp and mangrove forests we used expert-based estimates for the subtropics and tropics^44^.

For terrestrial anthropogenically modified habitat classes not already mapped by Copernicus, we relied on existing and novel human pressure datasets. Rural gardens were identified by (1) creating a boundary area of 500 m around urban land cover classes in the Copernicus data and (2) intersecting arable land cover within that boundary area with the “very small field size” category according to data on global field size distribution^45^. The 14.3 Plantations class is based on a novel global forest-management layer for the year 2015^46^ (available here^47^). To create this layer, separate labelling campaigns were run on sampled forested grid cells (according to Copernicus^34^ and Hansen forest cover change dataset^48^) for the tropical, temperate, boreal climate region using the GEO-WIKI platform^49^. Labellers were asked to classify the forest grid cells into several human-dominated forest classes. Finally the global forest management layer was created using a random-forest classifier applied on full PROBA-V time series for the year 2015^46^. We considered all replanted forests (rotation period longer than 20 years), short-rotation woody plantations, agroforestry and fruit plantations as plantation forests.

For pastureland we investigated several existing global pasture datasets for their suitability to serve as a pasture mask^50–52^, however we found them either too coarse or outdated, failing to highlight for instance the expansion of pastoral land in Brazil or unable to distinguish between different livestock management systems, for instance grazing in natural grassland versus man-made pastures. For the release (version 002) of the global habitat map we defined ‘Pastureland’ as grid cells with non-tree covered vegetation with at least 1 head per km^2^ of a grazing livestock-unit (LSU) on land climatically suitable for forest cover, that is, where trees would grow in the absence of grazing. To define the pasture mask we used the latest estimates of all grazing and browsing livestock (buffalo, cattle, goats, horses, sheep) from the gridded livestock density of the world dataset^53^ and converted them to LSU using region-specific conversion factors^54^. Originally forest-covered land was defined as those grid cells that are not in a grass, tundra, steppe or meadow defined ecoregion^37^ and which are in predominantly tree-covered climatic zones (Tropical, Temperate, Continental) according to the Köppen-geiger climate classification system^35^.

All aforementioned datasets were intersected to construct the global habitat map (Fig. 1) using a decision tree approach (see Supplementary Table 1 for coded rules). This was done in a hierarchical way, by first identifying the IUCN habitat class at level 1 i.e. Forest, Savanna, Shrubland, Grassland, Wetlands (inland), Rocky Areas, Deserts & Artificial habitats (but see Supplementary Table 1), followed by the level 2 classifications nested within the respective level 1 class through a decision tree (Fig. 1). The sequential order is important, with anthropogenically modified habitat classes always being mapped first and therefore masking all other ‘natural’ habitat classes. All calculations were implemented in Google Earth Engine (GEE), a cloud-based platform for remote sensing data processing^55^. Whenever the input layers differed in spatial resolution with Copernicus, we resampled those layers by taking the nearest-neighbor. The particular benefits of using GEE are computational speed (taking less than 4 h to create and export a new version), clear reproducibility and the ability to update the map easily as new or improved input layers become available. We provide a publicly accessible interface that lets users navigate the map and make all GEE code necessary to reproduce the map available (see code availability).

## Data Records

**Fig. 2:**
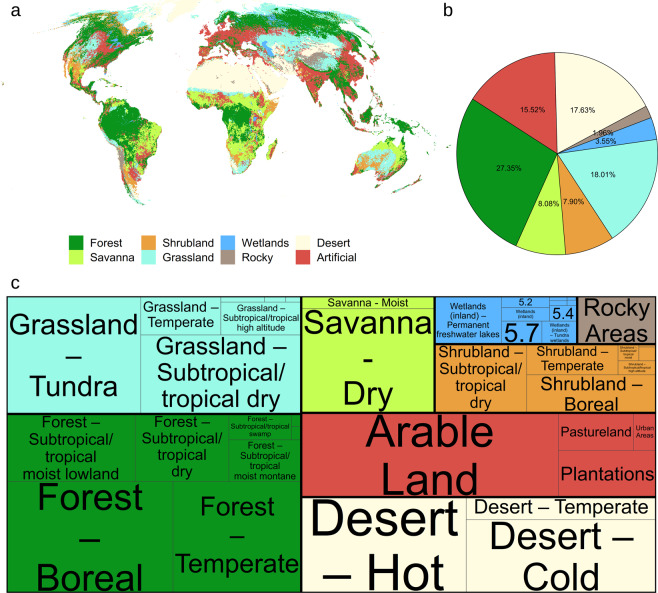
Distribution of IUCN habitat classes globally (**a**) Showing the Level 1 classification (coarsened to ~5 km for this visualization). (**b**) Proportion of global land area occupied by each Level 1 IUCN habitat class. (**c**) Tree map showing the most dominant IUCN habitat class at Level 2^16^ nested within the Level 1 classes. Colours as in (**a**) with classes scaled proportional to the land area. Level 2 classes with very long names were converted to their id number^16^, while small proportions might not be mapped.

The global habitat map for the year 2015 (version 003, Fig. 2) is made interactively available through Google Earth Engine (https://uploads.users.earthengine.app/view/habitat-types-map). As part of this manuscript, the map for Level 1 and Level 2 habitat classes has been made available on a public Zenodo repository at both the Copernicus ~100 m resolution and at fractional aggregated 1 km resolution^56^. The GEE code to recreate the map is available at (https://github.com/Martin-Jung/Habitatmapping). Asset data used in GEE are publicly readable and directly available from the original sources (see methods). The extent of global planted trees needed to reproduce the map has been made available here^47^. Users are advised to check the data repository for newer versions of both code and map, as we consider this product a “living map” that can be improved in the future pending better data availability. Soon, annual updates to Copernicus up to 2019 will be available^34^ and we also plan to create variants relying on the potential distribution of land cover and biomes^57^.

## Technical Validation

### Approach

Since the global habitat map was thematically created to match the IUCN habitats classification system, we mainly relied on existing, independently derived habitat information data to assess its accuracy. We relied on four different data sources for the validation, recognizing that none of them are without spatial bias^58^ and that it was not possible to find suitable validation data for all mapped habitat classes.

As a first source, we obtained occurrence records of all terrestrial ‘habitat specialist’ species (those considered to occur only in a single Level 2 habitat class according to IUCN Red List assessors) observed during 2005–2019 from the Global Biodiversity Information Facility (GBIF) and eBird (https://ebird.org/). We excluded observations outside the geographical range of a species (as mapped for IUCN Red List assessments), which result largely from misidentifications, vagrants or taxonomic mismatches. Only unique observations with a coordinate uncertainty smaller than 300 m (GBIF) or 30 m (eBird) were retained and we furthermore applied a conservative buffer of 300 m to all observations to account for positional errors. A total of 35152 points were used in this analysis associated with 828 habitat specialist species, 50% of which are birds, 22% reptile, 20% mammals and 8% amphibian species.

Second, we used data from Important Bird and Biodiversity Areas (IBAs,^22,59^) in which habitat specialist birds were known to occur. Specifically, available species checklists were used to identify those IBAs where a given habitat specialist bird species was known to occur, and we checked for the occurrence of that habitat within the IBA. In total, 2142 IBA polygons were used (mean area of 2584 km^2^ with 54% being smaller than 500 km^2^); however IBA polygons were tested multiple times for different habitats as IBAs can contain more than one habitat. Altogether, a total of 8181 IBA polygons (representing 758 habitat specialist bird species) were tested for the presence of the preferred habitat of species recorded there.

Third, we used species coordinates from the Projecting Responses of Ecological Diversity In Changing Terrestrial Systems (PREDICTS) database^60,61^, specifically for artificial habitat classes (14) that are usually not found as habitat specialism. Here we selected only those sites that were sampled after the year 2000, and furthermore we buffered each point by the sampling extent (measured in m). For artificial habitat classes in total, we used 1506 validation sites for ‘Arable Land’ (14.1), 1130 for ‘Pastureland’ (14.2), 732 for ‘Plantations’ (14.3) and 429 for ‘Urban Areas’ (14.5).

Fourth, we used the LACO-Wiki platform to visually assess the mapped habitat classes at level 2 using publicly available high-resolution satellite imagery^62^. Half the points were placed at random and half were stratified by habitat class, thus ensuring an even spatial and thematic spread globally. People familiar with the IUCN habitat classification system were then asked to label the respective point with a provided level 2 class. NDVI time series from Landsat and the PROBA-IV satellites as well as Flickr™ images taken in the vicinity were provided as guidance. An initial comparison of label agreement between experts reached a 81.5% agreement at level 1 and a 62.5% agreement at level 2. Given that many climatically similar classes at level 2 are very hard or impossible to distinguish visually from satellite imagery, we decided to use this data source only for habitats mapped at level 1 of the IUCN habitat type legend, plus for level 2 deserts, rocky and artificial habitats, which could be most robustly visually identified. In total, 2229 points were collected as part of this exercise.

We then calculated the match between all observed habitat classes (from the three data sources) and the predicted habitat class from the habitat map at ~100 m resolution (the resolution of the Copernicus land cover data) and at Level 1 and Level 2. We considered only habitat classes for validation for which at least 10 suitable independent validation records were available. For both levels and each dataset we calculated the overall accuracy and the balanced accuracy (to account for an imbalanced number of testing observations) per class and overall using the ‘caret’ package^63^.

In addition to the technical validation, we also presented the map to a number of regional experts to ask for their feedback on mapped classes, which helped to fine-tune the ruleset for creating the habitat map.

### Results

**Fig. 3:**
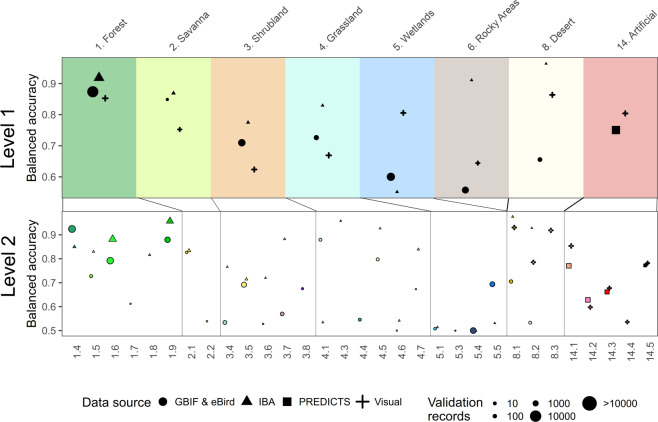
Validation results for the habitat map. Estimates of the balanced accuracy are shown for all habitats for which suitable validation data existed. Symbols indicate the validation data source, while point size shows the number of contributing records. The id corresponding to the specific IUCN habitat classes is indicated at the bottom and top of the figure. Colours match those of the online interactive interface (https://uploads.users.earthengine.app/view/habitat-types-map).

Across all considered datasets we found an overall accuracy of 0.62 for Level 1 and 0.55 for Level 2 of the mapped IUCN habitat classes. However there was a large disparity among validation datasets and number of classes. For the point records from GBIF/eBird/PREDICTS the overall accuracy at Level 1 was 0.55 (Level 2: 0.49), for the IBA data 0.91 (Level 2: 0.82), for the artificial habitats from the PREDICTS database 0.79 (Level 2: 0.45) and for the visual labeled sites at Level 1 0.60 (Level 2: 0.65). The average balanced accuracy across validation datasets was 0.76 ($\bar{+}$0.12 SD) at Level 1 and 0.72 ($\bar{+}$0.15 SD) at Level 2. We found the greatest balanced accuracy at Level 1 for ‘1. Forests’ with 0.88 and the lowest for ‘5. Wetlands’ with 0.65, while the difference in balanced accuracy between datasets was greatest between ‘6. Rocky areas’ and ‘8. Deserts’ (Fig. 3).

We were able to validate 29 of 48 habitat classes mapped at Level 2 of the IUCN habitat classification system (Fig. 3). Across datasets, the largest number of independent validation records was available for ‘1.6. Forest – Subtropical/tropical moist lowland’ (N = 8574) with the lowest being for ‘5.3. Wetlands (inland) – Shrub dominated wetlands’ and ‘4.3 Grassland – Subantarctic’ (both 12). For those habitat classes that could be validated at level 2 (Fig. 3), we found the highest balanced accuracy for ‘4.3 Grassland – Subantarctic’ (0.96), ‘8.3. Desert – Cold’ (0.92), 1.9. Forest – Subtropical/tropical moist montane’ (0.918) and ‘1.4. Forest – Temperate’ (0.88), and the lowest for, ‘5.3. Wetlands (inland) – Shrub dominated wetlands’ and ‘5.4. Wetlands (inland) – Bogs, marshes, swamps, fens, peatlands’ (all 0.5). The balanced accuracy for artificial habitat classes was found to be highest for ‘14.5 Urban Areas’ (0.81) and lowest for ‘14.4 Rural Gardens’ (0.54).

Overall, we stress that all of the validation data sources have characteristics that limit their utility for validating a habitat map, and the presented validation results should be interpreted with caution (see Usage notes).

## Usage Notes

### Validation interpretation

Independently validating a global habitat map is challenging. In this manuscript we mainly relied on biodiversity observations and sampling sites for validation, recognizing that doing so can be problematic for several reasons: (*a*) These observations can be spatially and taxonomically imprecise. For instance most vertebrates, particularly birds, are highly mobile and non-systematically collected observations (e.g. citizen-science initiatives like eBird) can occur in atypical habitats, for instance if a species is wrongly identified or a migrating bird recorded during passage. In addition, species occurrences obtained by direct, opportunistic observation tend to be biased towards accessible areas, therefore species tend to be observed at the margins of natural habitats rather than the core, which can result in attributing a record to the wrong habitat type. The fact that we had generally better accuracy for static sampling sites with observations performed by experts (IBAs and PREDICTS sites, Fig. 3) with larger sampling extent may confirm this assumption; (*b*) For the validation, we used records for those species which had only a single habitat listed as their preference, however it is quite likely that is an incomplete characterization of a species habitat preference. For instance, *Montifringilla nivalis* is said to exclusively occur in ‘6. Rocky Areas’, however within its range the species regularly occurs also in ‘4.4. Grassland – Temperate’ and ‘14.2 Pastureland’; (*c*) There can be errors in the assigned habitat preferences themselves. For instance, the endemic Japanese macaque (*Macaca fuscata*) is listed to occur exclusively in ‘1.6. Forest - Subtropical/Tropical Moist Lowland’^64^, although most of Japan (where the species is endemic, albeit widespread) is of temperate climate^35,37^. The fact that we were able to programmatically and quickly identify several incorrect habitat preferences in the IUCN Red List database suggests that mapping the IUCN habitat classes would help Red List assessors to code species’ habitat preferences more accurately swiftly, because it allows them to immediately visualize their mental model of a species’ habitats, and correct wrong or missing habitat preferences as well as validating their own assumptions about species ecology**;** (*d*) All biodiversity observations have obvious geographic and sampling biases, occurring predominantly in temperate regions and more accessible habitats and locations^58^. This is exemplified by the fact that we were not able to validate all mapped IUCN habitat classes directly, with boreal habitats missing entirely, while other habitat classes such as mangrove forests had very few records (Fig. 3).

In addition to the biodiversity observations and sampling sites, we also relied on a visual assessment of the habitat classes based on satellite imagery, which however also has limitations as a validation data source. Visual labeling of habitats is prone to human errors, depends on - often patchy distributed and outdated - high resolution satellite imagery coverage^65^ and is often not easily done for climatically similar classes. Indeed, particularly at level 2 some classes are very hard or impossible to distinguish visually even for experts, such as for instance ‘1.6. Subtropical/tropical moist lowland forest’ from ‘1.8 Subtropical/tropical swamp forest’.

The habitat map presented is an intersection of multiple existing datasets, each with its own uncertainty in the mapped classes. This uncertainty in the mapped input layers has only been explicitly mapped for land cover and climate data (Supplementary Figure 1), making it challenging to evaluate the influence of input data uncertainty on the mapped habitats^28^. We visually interpreted many of the mismatching species observations used for validation and often found fine-scale differences in land cover (e.g. ‘4.4. Grassland – Temperate’ to ‘3.4. Shrubland – Temperate’) to be the origin.

### Known limitations

The documentation of the IUCN habitat classification scheme is unfinished, with ~20% of all class descriptions lacking further elaboration^16^. In this study we aimed to follow the habitat classification system outlined by IUCN^16^ to facilitate links with other IUCN data, realizing that other - often more detailed - habitat classification systems exist at national scale^66,67^, using land cover and climate data of higher spatial and thematic resolution^19^. For instance, in an expert-based visual assessment of the habitat map we found that the most common error source were mistakes in the underlying global land cover data. Based on a precautionary principle and known limitations (see text file on the data repository), we recommend to use the habitat map at a coarsened resolution and supply fractional aggregated maps of each individual class at 1 km resolution with every release^56^.

Furthermore not all habitat types can be adequately mapped spatially, with some being only seasonally present^41^, having intra-annual sequences^68^ or being of ‘mixed’ nature, such as lightly-grazed savanna habitats which can be considered grassland, shrubland or forest depending on the vegetation cover. Other IUCN habitat classes are very hard to map spatially, such as ‘16. Introduced vegetation’. Better spatial information on other anthropogenic classes, such as sown pasture/rangelands, are also necessary to better represent this class in the global habitat map. In addition, four terrestrial IUCN habitat classes (four level 2 habitat classes) are not represented in the current version of the global habitat map, i.e. all marine habitats (habitat classes 9 to 13) as well as artificial aquatic habitats (15). We stress that the habitat map will be updated in the future as new or improved ancillary data become available, which will likely also help to improve many mapped classes.

### Suggestions to improve the IUCN habitat classification scheme

In the process of producing the first map of IUCN habitat classes, the potential for several improvements to the IUCN habitat classification system became apparent. Firstly, we suggest that additional classes could be added to represent managed forests other than plantations: specifically natural and semi-natural forests that are regularly logged, and recently cleared forests outside the tropics (category 14.6 is limited to heavily degraded or former forest within the subtropics and tropics); and mixed classes of forest/shrubland/grassland, for instance for ‘Temperate open woodland’. For anthropogenic IUCN classes, we suggest that, besides the existing ‘14.2 Pastureland’ class, another class ‘14.7 Rangeland’ could be established, that explicitly relates to anthropogenically grazed natural grasslands in arid regions, like the Kalahari or Western Australia Shrublands^37^ and rangelands in the Chaparral. The definition of ‘14.2 Pastureland’ is limited to intensively managed ‘fertilized or re-seeded permanent grasslands, sometimes treated with selective herbicides, with very impoverished flora and fauna’^16^ which is an extremely small fraction of all areas that are grazed by livestock. In addition, many existing habitat classes without defined descriptions require additional documentation to make it feasible to map them spatially.

## Supplementary information

Supplementary Figure 1

Supplementary Table 1

## Data Availability

All programming code necessary to reproduce the map in Google Earth Engine is supplied together with the data (see Data records) and on https://github.com/Martin-Jung/Habitatmapping.
