# Supplementary material for: A global map of terrestrial habitat types
Source: Sci Data. 2020 Aug 5;7:256. doi: 10.1038/s41597-020-00599-8 (PMC7406504; doi:10.1038/s41597-020-00599-8)
Supplement: Supplementary file 1 — Supplementary Figure 1 [file 41597_2020_599_MOESM1_ESM.pdf]

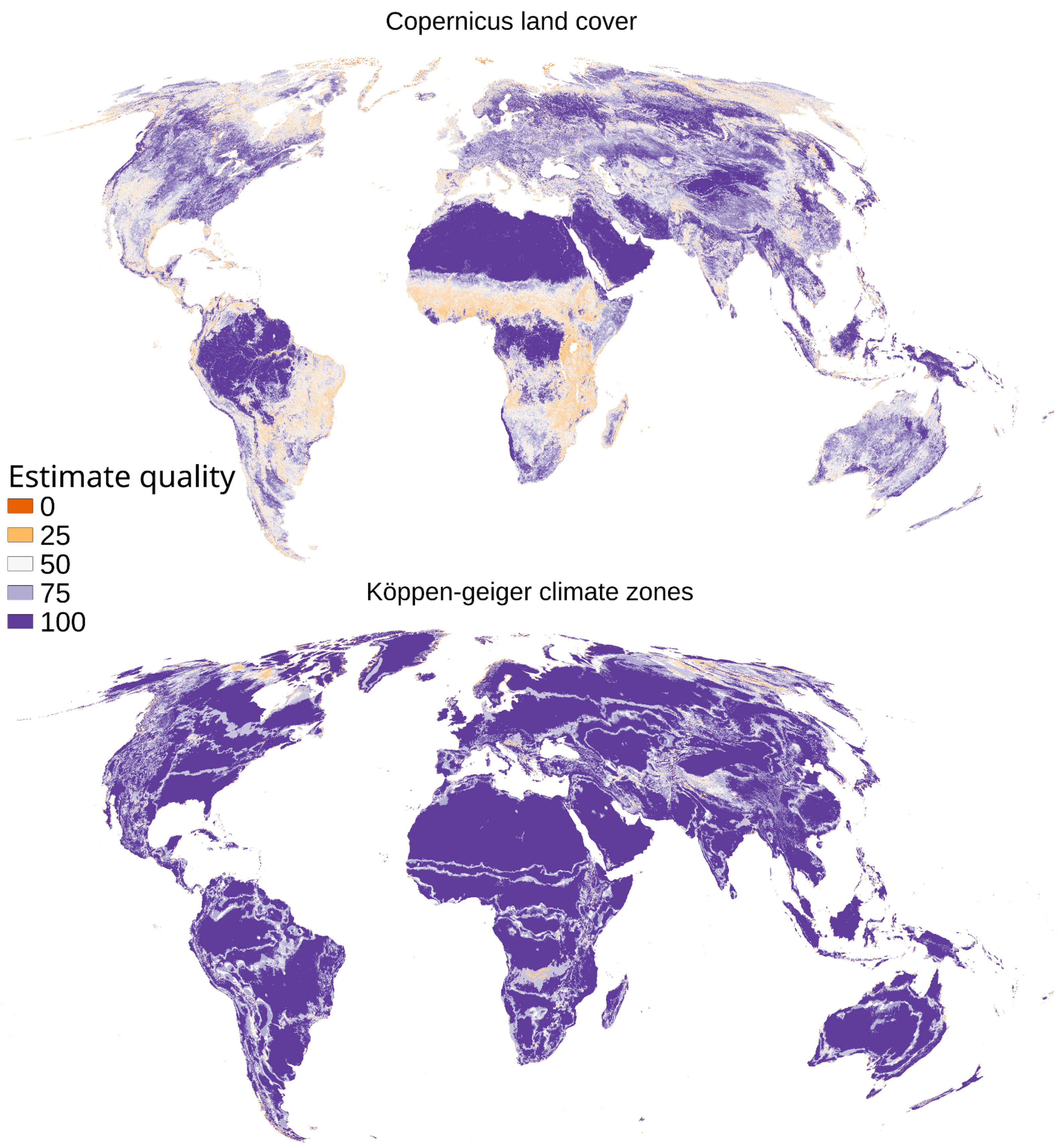

Supplementary Figure 1: Estimate quality of the used land cover and climate data. Estimate of the quality (inverse of uncertainty) of both the Copernicus land cover data [34] and the Köppen-geiger climate zones [35].
